# Community health workers’ knowledge, attitudes and practices in the detection of respiratory symptomatic patients

**DOI:** 10.1590/0034-7167-2024-0610

**Published:** 2025-12-08

**Authors:** Ana Flavia de Oliveira Ribeiro, Laura Maria Vidal Nogueira, Erlon Gabriel Rego de Andrade, Ivaneide Leal Ataíde Rodrigues, Ingrid Bentes Lima, Breno Augusto Silva Duarte

**Affiliations:** I Universidade do Estado do Pará. Belém, Pará, Brazil

**Keywords:** Health Knowledge, Attitudes, Practice, Community Health Workers, Signs and Symptoms, Respiratory, Tuberculosis, Primary Health Care., Conocimientos, Actitudes y Práctica en Salud, Agentes Comunitarios de Salud, Signos y Síntomas Respiratorios, Tuberculosis, Atención Primaria de Salud.

## Abstract

**Objectives:**

to analyze the association between community health workers’ knowledge, attitudes, and practices regarding respiratory symptomatic patient detection in Primary Health Care.

**Methods:**

a cross-sectional study conducted in Family Health Units in Santa Izabel do Pará, Brazil, with a census sample of 129 community health workers. A questionnaire based on the Advocacy, Communication, and Social Mobilization for TB Control guide was used to survey knowledge, attitudes, and practices. Descriptive statistics and chi-square test were used, with a 95% significance level (p≤0.05).

**Results:**

the predominant participants were female (n=96/74.4%), aged 40-49 years (n=54/41.9%), with complete high school education (n=86/66.7%), Catholic (n=65/50.4%), brown skin color/race (n=109/84.5%), and 20 years of experience (n=55/42.6%). Associations between knowledge and attitudes (p=0.2582) and between knowledge and practices (p=0.8973) were not statistically significant.

**Conclusions:**

workers’ attitudes and practices in the search for respiratory symptomatic patients were not determined by their level of knowledge about tuberculosis.

## RESUMEN

**Objetivos:**

analizar la asociación entre conocimientos, actitudes y prácticas de los agentes de salud comunitarios en la detección de pacientes con síntomas respiratorios en Atención Primaria de Salud.

**Métodos:**

estudio transversal realizado en Unidades de Salud Familiar de Santa Izabel do Pará, Brasil, con una muestra censal de 129 agentes de salud comunitarios. Se utilizó un cuestionario basado en la guía *Advocacy, Communication and Social Mobilization for TB Control* para relevar conocimientos, actitudes y prácticas. Se emplearon estadísticas descriptivas y la prueba de chi-cuadrado, con un nivel de significancia del 95% (p≤0,05).

**Resultados:**

los participantes predominantes fueron mujeres (n=96/74,4%), con edades comprendidas entre 40 y 49 años (n=54/41,9%), con educación secundaria completa (n=86/66,7%), católicos (n=65/50,4%), de piel morena (n=109/84,5%) y con 20 años de experiencia (n=55/42,6%). Las asociaciones entre conocimientos y actitudes (p=0,2582) y entre conocimientos y prácticas (p=0,8973) no fueron estadísticamente significativas.

**Conclusiones:**

las actitudes y prácticas de los agentes en la búsqueda de pacientes con síntomas respiratorios no estuvieron determinadas por su nivel de conocimientos sobre tuberculosis.

## INTRODUCTION

Controlling tuberculosis (TB) in the community is becoming increasingly challenging, due to health teams’ limited investment in identifying respiratory symptomatic patients (RSPs) of interest for timely diagnosis^(1-3)^. In this regard, community health workers (CHWs) are crucial, as they carry out their work activities in the territory, in direct contact with people^(4,5)^.

Caused by *Mycobacterium tuberculosis* or Koch’s bacillus, TB preferentially affects the lungs, being called pulmonary TB, but it can also affect other organs, characterizing extrapulmonary TB^(1)^. It constitutes a significant public health problem, as it is the leading cause of death from a single infectious agent globally. However, during the health emergency caused by the COVID-19 pandemic, TB has become the second leading cause of death, behind only COVID-19. In a context marked by health determinants and vulnerabilities, this highlights that efforts to control TB are hampered by cultural, economic, political, social, clinical, and healthcare factors^(6,7)^.

Adherence to treatment is vital to achieving a cure and interrupting the chain of transmission^(6)^. However, as official data states, effective control is still far from reality, as it is estimated that, worldwide, in 2023, the disease affected 10.8 million people and caused 1.25 million deaths^(7)^. Analyzing the national territory, it can be seen that, in the same year, 80,012 people fell ill and, in 2022, 5,845 died from TB, resulting in incidence and mortality rates equal to 37.0 cases per 100,000 inhabitants and 2.72 deaths per 100,000 inhabitants. In the state of Pará, these indicators worsened, as, in 2023, 4,293 people fell ill, and in 2022, 341 died, with rates of 48.8 cases per 100,000 inhabitants and 3.9 deaths per 100,000 inhabitants^(8)^.

In 2014, during an assembly promoted by the World Health Organization (WHO), the End TB Strategy was approved, with the aim of tackling the disease and eliminating it by 2035^(9)^. Given Brazil’s experience with the Brazilian Health System (In Portuguese, *Sistema Único de Saúde* - SUS) implementation and operation, and the Brazilian Tuberculosis Research Network’s (In Portuguese, *Rede Brasileira de Pesquisas em Tuberculose* - Rede-TB) contributions, its participation is crucial to achieving the global target. Therefore, based on this strategy, the Brazilian National Plan to End TB as a Public Health Problem has the following goals by 2035: reducing the incidence rate to less than ten cases per 100,000 population; and reducing the TB mortality rate to less than one death per 100,000 population^(2)^.

Case identification occurs through the investigation of people suspected of having the disease, configuring systematic tracking, for which tests or other simple and quick procedures must be used^(3)^. This screening is done through active screening, a process of identifying people with persistent coughs who have not yet accessed healthcare services and who, based on their symptoms, may have pulmonary or laryngeal TB. It is necessary to assess cough duration and consider individuals’ risk of illness and access to formal care, with the aim of referring them for diagnostic testing^(10)^.

RSPs are defined as those with a cough lasting three weeks or more. It is important to note that, in active screening, cough duration should be taken into account in the population being investigated. It is essential to identify RSPs so that it can be diagnosed and promptly treated, thus interrupting the chain of transmission^(3,11)^. In this context, CHWs are social actors who play an important role in health practices related to the search for RSPs, as they are routinely in the community to maintain the relationship between users and healthcare services^(4)^.

Strategies for seeking out RSPs must be well planned, especially within the Family Health Strategy (FHS), during home visits, occasions in which professionals must talk to users and share the necessary guidance to examine them carefully^(12)^.

Considering the potential contribution of studies on knowledge, attitudes, and practices (KAPs) to consolidate programs and actions aimed at preventing and controlling the disease, and taking into account the importance and multiplicity of CHWs’ responsibilities in this context, it is understood that these professionals’ knowledge and the way they act are factors that can interfere with the quality and effectiveness of their actions and, consequently, in the disease indicators at the local level^(4,5,13)^.

Studies on KAPs use a specific method and are designed to measure what people know, feel, think, and how they behave in relation to a given topic, in this case, TB^(13,14)^. This assessment is made considering that they present varied knowledge, different attitudes, and practices adopted over time^(15)^.

These studies help plan, execute, and assess health actions, enabling the identification of problems, needs, barriers, and solutions to improve the quality and accessibility of services. This context allows managers to set goals and priorities, estimate costs and available resources, and promote professional training. Furthermore, they contribute to producing data on diseases and conditions to provide managers and their teams with the information needed to formulate new strategies, helping to identify factors that influence certain behaviors, which are often not consciously understood by most people^(3,13,15)^.

Conceptually, knowledge refers to understanding of any subject; attitude relates to feelings and preconceived ideas; and practice represents actions that arise from how knowledge and attitude are expressed. Understanding the levels of KAP favors the process of knowledge creation, increasing the possibilities for services to adapt and meet individual and collective needs^(14)^.

Considering the scientific and social relevance of this topic for the health field, especially public health, investments in this field can reveal CHWs’ mindset and behaviors regarding TB and RSPs, contributing to strengthening strategies for detecting symptomatic patients and identifying new cases, for which timely treatment should be prescribed.

Therefore, the object of this study was defined as CHWs’ KAPs regarding RSPs detection. To investigate this, the following questions were formulated: what are the CHWs’ KAPs regarding RSPs detection? Are these elements associated with CHWs in detecting RSPs in Primary Health Care (PHC)? Therefore, the initial hypothesis was that KAPs interfere with RSP detection.

## OBJECTIVES

To analyze the association between community health workers’ knowledge, attitudes, and practices regarding respiratory symptomatic patient detection in Primary Health Care.

## METHODS

### Ethical aspects

The guidelines of Resolution 466/2012 of the Brazilian National Health Council/Ministry of Health were followed, obtaining institutional approval from the Municipal Health Department of Santa Izabel do Pará and approval from the Research Ethics Committee of the undergraduate nursing program at the *Universidade do Estado do Pará*, issued in February 2022. Participants read and signed the Informed Consent Form (ICF), expressing their voluntary acceptance. To protect the confidentiality of their identities, they were assigned alphanumeric codes consisting of the acronym CHW followed by a sequential Arabic numeral.

### Study design and place

This is a cross-sectional study guided by STrengthening the Reporting of OBservational studies in Epidemiology (STROBE)^(16)^.

It was carried out in 18 Family Health Units (FHU) and in a Riverside Family Health Unit (RFHU) in the municipality of Santa Izabel do Pará, state of Pará, Brazil, one of the eight municipalities that make up the Metropolitan Region of Belém. It is approximately 46.6 km from Belém, the state capital^(17)^, with a territorial area of 717.662 km^2^ and an estimated population of 73,019 inhabitants, according to the current Brazilian Institute of Geography and Statistics Demographic Census^(18)^. It is composed of several villages and three districts: Americano, Caraparu and Santa Izabel (municipal headquarters)^(19)^.

These units offer PHC services through multidisciplinary teams made up of nurses, physicians, dentists, nursing technicians, oral health assistants and a variable number of CHWs (around seven or eight per unit)^(20)^.

### Population and selection criteria

A total of 129 CHWs participated, accounting for 90.2% of the 143 who worked in the municipality during the data collection period, constituting a census sample. We chose to include those who were fully exercising their functions and had been working professionally for at least three months. Two (1.4%) were excluded because they were on vacation, leave, or otherwise absent from their duties, regardless of the cause, and 12 (8.4%) refused to participate, totaling 14 (9.8%) losses among eligible participants. Furthermore, there were no dropouts.

### Study protocol

The first author visited the units to explain the project to the nurse coordinators of family health teams, requesting their support in implementing data collection. Thus, at each unit, a meeting was scheduled with CHWs, held in a private room, to present the proposal, clarify any questions, and invite them to participate. Those who accepted remained in the room to schedule an individual meeting, which took place privately at the units where they worked, ensuring their privacy and without interfering with care routine.

To avoid possible biases, meetings were led by the author, a master’s student in nursing, duly trained in scheduled meetings for guidance activities and in regular meetings of a research group based at the authors’ institution. They began with ICF presentation to detail the objective, procedures, risks, and benefits of the research, aiming to obtain formal acceptance from participants. Subsequently, an instrument formulated by the authors was applied, based on the questions from the Advocacy, Communication, and Social Mobilization for TB Control guide for KAPs research on TB, developed by the WHO^(13)^, and on the guidelines of the booklet on TB for CHWs, published by the Ministry of Health^(5)^, a procedure similarly adopted in another study^(21)^.

In the format of a self-administered questionnaire, this instrument consisted of 37 questions, distributed in four sections, of which section I presented ten questions about CHWs sociodemographic and occupational characteristics, whose alternatives were multiple choice with a single answer. These questions were: 1) “How old are you?”; 2) “What is your sex?”; 3) “What is the highest level of education you have completed?”; 4) “What is your religion?”; 5) “What is your color/race?”; 6) “What is your workplace/health unit?”; 7) “How long have you been working as a CHW?”; 8) “What is the total number of users you follow?”; 9) “How many people with TB do you follow?”; 10) “When was the last time you received training on TB?”.

Regarding general knowledge about TB, section II consisted of 14 closed-ended questions: 1) “Is TB caused by the *Mycobacterium tuberculosis* bacterium?”; 2) “Is TB a serious disease?”; 3) “Is TB a problem in your country/region?”; 4) “Can shaking hands and sharing dishes, cutlery, and towels transmit TB?”; 5) “Is TB transmitted through the air when an infected person coughs or sneezes?”; 6) “Among the TB signs and symptoms are a cough that lasts more than three weeks, coughing up blood, weight loss, fatigue, chest pain, and fever in the late afternoon?”; 7) “Is pulmonary TB diagnosis primarily made through smear microscopy?”; 8) “Is smear microscopy performed by collecting two sputum samples, one at the time the person with respiratory symptoms is identified and the other the following morning?”; 9) “Is covering the mouth and nose when coughing or sneezing one of the instructions that should be given to patients with TB?”; 10) “Is TB curable?”; 11) “Is TB treatment available through the SUS?”; 12) “Does TB treatment last six months, with two months corresponding to the attack phase and the other four months to the maintenance phase?”; 13) “Should all people with TB take a rapid HIV test?”; 14) “Is a person with respiratory symptoms someone who has a cough lasting three weeks or more?”.

It presented the alternatives “strongly agree”, “agree”, “undecided”, “disagree”, and “strongly disagree”. Based on a Likert scale, it had a final score with three knowledge categories: “adequate/good”; “regular”; and “inadequate/insufficient”. The “adequate/good” category was assigned when the CHW answered “agree” or “strongly agree” to at least ten questions; the “regular” category when they answered “agree” or “strongly agree” to five to nine questions; and the “inadequate/insufficient” category when they answered “agree” or “strongly agree” to fewer than five questions.

About attitudes in the search for RSPs, section III included five closed questions: 1) “When I identify a patient with a cough for three weeks or more, do I investigate TB?”; 2) “Do I refer RSPs for consultation with a nurse and/or physician?”; 3) “Do I refer RSPs for sputum smear microscopy?”; 4) “Do I record the active search for RSPs in the Home Visit Form or in the e-SUS PHC Territory application (if using a tablet)?”; 5) “Do I report the RSPs to a nurse so that they can record them in the Respiratory Symptomatic Patient Book?”.

It presented the alternatives “strongly agree”, “agree”, “undecided”, “disagree”, and “strongly disagree”. It had a final score with three attitude categories: “adequate/good”; “regular”; and “inadequate/insufficient”. The “adequate/good” category was assigned when the CHW answered “agree” or “strongly agree” to four or five questions; the “regular” category when they answered “agree” or “strongly agree” to two or three questions; and the “inadequate/insufficient” category when they answered “agree” or “strongly agree” to none or only one question.

Concerning practices in the search for RSPs, section IV included eight multiple-choice questions with a single answer: 1) “Do you question the cough presence and duration during home visits?”; 2) “How do you advise the population about airborne transmission of TB?”; 3) “How do you advise the population about TB signs and symptoms?”; 4) “How do you advise the population about the preventive measures that can be adopted?”; 5) “Do you prioritize the active search for RSPs in your daily life?”; 6) “Do you teach how to collect material for sputum smear microscopy?”; 7) “Do you notify nurses when you find someone with respiratory symptoms?”; 8) “Do you question the cough presence and duration in contacts of people with TB?”.

It presented a final score with three practice categories: “adequate/good”; “regular”; and “inadequate/insufficient.” The “adequate/good” category was assigned when the CHW answered seven or eight questions correctly; the “regular” category, when they answered four, five, or six questions correctly; and the “inadequate/insufficient” category, when they answered none, one, two, or three questions correctly.

Data were collected between April 6 and October 7, 2022, a period preceded by a pre-test of the instrument, carried out in the penultimate week of March of that year, with ten CHWs working in Benevides, a neighboring municipality with similar economic and sociodemographic characteristics to Santa Izabel do Pará, who were not included in the study sample. The pre-test helped to adapt the questionnaire and estimate its administration time. It should be noted that, although it was not validated with experts and/or the target audience, this instrument allowed the study objective to be achieved.

### Analysis of results and statistics

Data were organized into Microsoft Office Excel^®^ (version 2016) spreadsheets for descriptive statistical analysis, identifying the absolute and relative frequencies of sociodemographic and occupational profiles and characteristics related to CHWs’ KAPs. To analyze the association between knowledge level and attitudes and practices, the chi-square test was applied using BioEstat software (version 5.3). A significance level of 95% and a p-value ≤ 0.05 were considered. The decision to use both software programs aligns with the research group’s routine and work practices.

## RESULTS

Among the 129 participants, female sex (n=96; 74.4%), age group from 40 to 49 years (n=54; 41.9%), education level inherent to complete high school (n=86; 66.7%), Catholic religion (n=65; 50.4%) and brown color/race (n=109; 84.5%) predominated, as shown in Table 1.

**Table 1:** Participant sociodemographic characteristics (N=129), Belém, Pará, Brazil, 2022

Characteristics	n	%
Sex		
Male	33	25.6
Female	96	74.4
Age group		
Under 30	10	7.8
30 to 39	29	22.5
40 to 49	54	41.9
50 or older	36	27.9
Education		
Complete elementary school	3	2.3
Complete high school	86	66.7
Complete higher education	39	30.2
Not provided	1	0.8
Religion		
Catholic	65	50.4
Evangelical	49	38.0
No religion	10	7.8
Other	5	3.9
Color/race		
White	7	5.4
Brown	109	84.5
Black	13	10.1

In relation to occupational characteristics, it was found that 55 (42.6%) had been working professionally for more than 20 years and that the number of people with TB monitored by CHWs was variable, with a prevalence of those who, during data collection, reported not monitoring people with this disease (n=102; 79.1%). The majority (n=78; 60.5%) received training on TB more than five years ago, and only five (3.9%) received it in the last year, it is important to highlight that eight (6.2%) stated that they had never received it (Table 2).

**Table 2:** Participant occupational characteristics (N=129), Belém, Pará, Brazil, 2022

Characteristics	n	%
Length of professional experience		
Under five years	6	4.7
Between five and nine years	20	15.5
Between ten and 14 years	25	19.4
Between 15 and 19 years	23	17.8
20 years or older	55	42.6
Number of people with TB monitored by CHWs		
None	102	79.1
One	21	16.3
Two	3	2.3
Three	0	0.0
Four	1	0.8
Five	0	0.0
More than five	2	1.6
Latest TB training		
One year ago	5	3.9
Two years ago	2	1.6
Three years ago	5	3.9
Four years ago	16	12.4
Five years ago	15	11.6
More than five years ago	78	60.5
Never received training	8	6.2

*CHW - community health worker; TB - tuberculosis.*

For general knowledge about TB, 121 (93.8%) presented knowledge allocated to the “adequate/good” category, with no “inadequate/insufficient” category being identified. Similarly, the majority (n=113; 87.6%) presented attitudes allocated to the “adequate/good” category, with no “inadequate/insufficient” category being identified. Furthermore, 54 (41.9%) presented practices allocated to the “adequate/good” category, and 17 (13.2%) to the “inadequate/insufficient” category (Table 3).

**Table 3:** Measurement of participants’ knowledge, attitudes, and practices regarding tuberculosis and respiratory symptomatic patients (N=129), Belém, Pará, Brazil, 2022

Section	n	%
Knowledge		
Adequate/good	121	93.8
Regular	8	6.2
Attitude		
Adequate/good	113	87.6
Regular	16	12.4
Practice		
Adequate/good	54	41.9
Regular	58	45.0
Inadequate/insufficient	17	13.2

The statistical association between knowledge and attitudes was not significant (p=0.2582), as well as the association between knowledge and practices (p=0.8973), allowing us to state that CHWs’ attitudes and practices in the search for RSPs were not determined by the level of knowledge they had about TB.

The proportion of CHWs with adequate/good knowledge and who had adequate/good attitudes (n=107; 88.4%) was close to that of those who had average knowledge and presented adequate/good attitudes (n=6; 75.0%). The same was observed in relation to practices, as the proportion of CHWs with adequate/good knowledge and who had adequate/good practices (n=50; 41.3%) was also close to that of those who had average knowledge and presented adequate/good practices (n=4; 50.0%), as shown in Table 4.

**Table 4:** Association between knowledge, attitudes and practices of participants (N=129), Belém, Pará, Brazil, 2022

Attitude	Knowledge				*p* value^*^
	Adequate/good	%	Regular	%	
Adequate/good	107	88.4	6	75.0	0.2582
Regular	14	11.6	2	25.0	
**Practice**					
Adequate/good	50	41.3	4	50.0	0.8973
Regular	55	45.5	3	37.5	
Inadequate/insufficient	16	13.2	1	12.5	

* *chi-square test.*

## DISCUSSION

The results indicate, above all, that CHWs’ knowledge and attitudes were considered adequate, in a context in which practices were mostly regular, demonstrating that their attitudes and practices were not aligned with their knowledge. Thus, the study hypothesis was refuted, since the KAPs did not interfere with RSPs detection.

Most individuals demonstrated adequate knowledge of various aspects of TB, including the etiological agent, severity, magnitude in the country/region, mode of transmission, signs and symptoms, diagnosis, treatment, and the need for testing to diagnose possible Human Immunodeficiency Virus (HIV) infection, demonstrating knowledge of the concept of RSPs. Adequate attitudes were those related to identifying the RSPs of interest for TB diagnosis, coughing for three weeks or more, referring RSPs for consultation with a nurse and/or physician, referring for sputum smear microscopy, recording the active search, and recording in the Respiratory Symptomatic Patient Book.

In turn, practices were considered regular, highlighting that the adequate knowledge and attitude profile was not reflected in CHWs’ actions within the community. It is possible to infer that this occurred because CHWs were familiar with the disease and the necessary attitudes to practice the profession, but unaware of their responsibilities in relation to it. This fact is possibly explained by three factors: differences in health knowledge among professionals working in a given context; differing attitudes among them; and practices acquired over time^(22)^.

Among sociodemographic and occupational characteristics, the following stood out: female gender, age 40 or older, high school education, Catholic religion, self-identified brown skin color, 20 or more years of experience in the role, and those who reported having received specific TB training for more than five years. The predominance of females in this population has also been identified in various studies as has age around 40 or older^(4,21,23,24)^. However, in another study, CHWs received at least one training in the last two years, and sought information about TB from coworkers and from pamphlets, internet texts, radio or television^(21)^.

It is understood that the absence or limited availability of continuing education actions on TB has an impact on the level of knowledge of CHWs, since, in their daily health work, they face many challenges in responding to the territory demands, requiring contextualized information to offer qualified conduct^(25)^.

In this context, they are essential actors in maintaining epidemiological surveillance, which necessarily includes the active search for RSPs. Therefore, educational initiatives aimed at CHWs constitute important measures to aggregate knowledge and contribute to the development of critical-reflective thinking, aiming to enhance the ability to articulate technical knowledge with popular knowledge, which circulates strongly among CHWs and in the community. This is relevant because the ability to articulate different knowledge, especially in the community context, is crucial for identifying RSPs^(4,26)^.

In addition to training, it is important to highlight the time spent working as a CHW as an important element, which is essential for strengthening bonds in close contact with human groups, but which can also provide an opportunity to aggregate, solidify or reframe knowledge acquired throughout professional practice^(26)^.

Although, in this study, the associations between knowledge and attitudes and between knowledge and practices did not reach statistical significance, it is important to consider that, to some extent, knowledge can influence attitudes and practices on a given topic, as indicated in the scientific literature^(4,27)^. Thus, erroneous practices in the professional context, which result from knowledge gaps, can compromise the achievement of objectives and goals. In the case of active search for RSP, they can interrupt the early detection of new TB cases and, thus, maintain the chain of transmission^(28)^.

Regarding treatment, it was identified that not everyone knew that duration, in PHC units’ routine, is six months, with the first two corresponding to the attack phase and the other four, to the maintenance phase, as recommended by the Ministry of Health^(3)^. Similarly, some were unaware of the ministerial recommendation regarding the need for rapid testing to detect or rule out HIV infection in people with TB^(3)^. Although not the majority, it is understood that, since the work of CHWs occurs individually within the territory, i.e., each CHW is responsible for monitoring an assigned area, it is necessary to standardize knowledge to achieve satisfactory results throughout the municipality^(4)^.

A study conducted with CHWs in the Eastern Cape Province, South Africa, identified misconceptions related to the cause of TB, associating it with smoking and cold weather, which constitutes a worrying scenario, given the role these professionals play on the front line of sharing information about the disease throughout the community, which can result in delays in identifying cases^(23)^. In another study, carried out in the ten districts of Lesotho, also located on the African continent, CHWs’ knowledge about TB was classified as inadequate, as they were unaware of basic information, such as signs and symptoms, and what should be valued after discharge from medication at the end of treatment, a scenario in which the authors reflected on the need to offer continuing education actions, although participants did not relate inadequate knowledge to the absence of a systematic training program^(24)^.

It is assumed that the long professional experience, evidenced in the majority of participants in this study, probably contributes to improving knowledge and encouraging the sharing of information among CHWs^(14)^. This is attributed to the satisfactory knowledge about TB, identified even among those who reported not having received training. However, it is necessary to monitor to avoid misinformation reproduction about the disease transmission or any other aspects, since distorted understanding can lead to harmful practices^(11,28)^.

Among the five questions about attitudes toward RSPs, the highest number of correct answers related to referral for consultation with a nurse and/or physician, without considering the need for prompt referral for sputum smear microscopy, reinforcing the view that only nurses and/or physicians are responsible for certain actions. The TB booklet for CHWs, published by the Ministry of Health^(5)^, states that these actors can also refer RSPs for sputum smear microscopy, as long as the municipality allows it through a protocol.

The majority agreed with the attitude of reporting to nurses the existence of RSPs in their assigned area, understanding it as adequate, as it allows nurses to register in the Respiratory Symptomatic Patient Book, although, according to participants, such action does not materialize in the municipality where this study was carried out.

Regarding public education regarding airborne transmission of TB, its signs and symptoms, and preventive measures, most respondents reported frequently conducting health education activities in the community. However, some respondents responded that they only provide guidance when requested, while others responded that nurses should provide guidance during consultations. It is worth noting that, according to the Ministry of Health, this guidance is part of CHWs’ responsibilities^(5)^, as well as other members of the multidisciplinary team, such as nurses, physicians, and nursing technicians^(2,3)^.

Everyone agreed with the practice related to prioritizing the active search for RSPs, as it is important to identify TB cases early. However, some stated they had other, more pressing needs to address, and some stated they only seek out outreach when they have free time, suggesting that perhaps some CHWs did not yet understand the importance of active community outreach.

In the practice of providing guidance on the good collection of material for sputum smear microscopy, CHWs’ understanding is that this is a responsibility of nurses and/or professionals from the laboratory where it will be performed, omitting an important function, because, although nurses have a relevant role in this process, CHWs are also responsible for these guidelines^(5)^.

In line with these results, another study found that most CHWs reported actively seeking out RSPs during home visits and referring suspected cases to FHS^(21)^, as standardized by the Ministry of Health^(5)^. However, only a small proportion reported requesting sputum smear microscopy and providing guidance on sputum collection to RSPs, suggesting knowledge gaps among these professionals and/or problems related to the structure or organization of TB control actions at the local level^(21)^.

Contact testing practices were neglected, as CHWs only expressed this approach when contacts reported something suspicious, often considering such an investigation unnecessary. However, the literature demonstrates that contact testing is a priority due to the risk of illness among contacts infected with *Mycobacterium tuberculosis*, who constitute potential sources of infection and maintain the chain of transmission in the community^(3,12)^. Therefore, contact surveillance actions require special attention from municipal managers, in order to encourage health teams and provide the necessary conditions to systematically carry out this activity^(2)^.

It is important to emphasize that CHWs’ practices must be monitored by nurses in order to assess their knowledge and define a possible targeted and contextualized educational plan, since, with adequate knowledge, the possibilities of implementing effective strategies increase^(26)^. In health work, educational activities constitute a proposition defended in the academic-scientific environment, since professionals must be regularly updated to respond satisfactorily to human groups’ health needs^(22)^.

### Study limitations

As a limitation, it is acknowledged that the study was conducted in only one municipality in Pará, demonstrating a specific local-regional reality. However, its results can support comparative analyses with other settings, investigating KAPs among CHWs and other actors.

### Contributions to nursing, health, or public policy

By highlighting the sociodemographic and occupational characteristics and data inherent to CHWs’ KAPs, this study contributes to the understanding of this reality by actors in the academic-scientific community and public administration working in the fields of health and nursing, proposing and implementing new strategies to help transform it, a context in which public policies targeting professionals stand out.

## CONCLUSIONS

It was found that CHWs’ KAPs did not present a significant statistical association with each other, but they brought important reflections about the performance of these professionals and the functioning of PHC services to control TB in the municipality investigated, since, despite the predominance of adequate knowledge and attitudes, practices were considered regular, configuring an important divergence.

It should be noted that, to effectively control the disease, it is necessary to expand or strengthen the level of knowledge of professionals to qualify and reorient their practices in the care network, aiming to improve actions in healthcare services and clinical management, considering that their KAPs can improve or compromise the quality of control strategies developed in PHC.

In view of this, it is recommended that further studies be carried out on the topic, especially with CHWs in different scenarios in the regional and national territories, enabling, on the one hand, a better understanding of the factors that determine their KAPs and, on the other, a comparative analysis of the data that will result from these studies.

## Data Availability

The research data are available within the article.
