# Supplementary material for: Community health workers’ knowledge, attitudes and practices in the detection of respiratory symptomatic patients
Source: Rev Bras Enferm. 2025 Dec 8;78(6):e20240610. doi: 10.1590/0034-7167-2024-0610 (PMC12707531; doi:10.1590/0034-7167-2024-0610)
Supplement: Translation

# Translation: Conhecimentos, atitudes e práticas de agentes comunitários de saúde na detecção de sintomáticos respiratórios

**DOI:** 10.1590/0034-7167-2024-0610pt

**Authors:** Ana Flavia de Oliveira Ribeiro, Laura Maria Vidal Nogueira, Erlon Gabriel Rego de Andrade, Ivaneide Leal Ataíde Rodrigues, Ingrid Bentes Lima, Breno Augusto Silva Duarte

**Affiliations:** I Universidade do Estado do Pará. Belém, Pará, Brasil

**Keywords:** Conhecimentos, Atitudes e Prática em Saúde, Agentes Comunitários de Saúde, Sinais e Sintomas Respiratórios, Tuberculose, Atenção Primária à, Saúde.

## Abstract

**Objetivos:**

analisar a associação entre conhecimentos, atitudes e práticas de agentes comunitários de saúde sobre a detecção de sintomáticos respiratórios na Atenção Primária à Saúde.

**Métodos:**

estudo transversal, realizado em Unidades de Saúde da Família de Santa Izabel do Pará, Brasil, com amostra censitária de 129 agentes comunitários de saúde, empregando questionário baseado no guia *Advocacy, Communication and Social Mobilization for TB Control* para pesquisas sobre conhecimentos, atitudes e práticas. Utilizaram-se estatística descritiva e teste qui-quadrado, com significância de 95% (p≤0,05).

**Resultados:**

predominaram sexo feminino (n=96/74,4%), faixa etária de 40-49 anos (n=54/41,9%), ensino médio completo (n=86/66,7%), religião católica (n=65/50,4%), cor/raça parda (n=109/84,5%) e tempo de atuação maior ou igual a 20 anos (n=55/42,6%). Associações entre conhecimentos e atitudes (p=0,2582) e entre conhecimentos e práticas (p=0,8973) não foram estatisticamente significantes.

**Conclusões:**

atitudes e práticas dos agentes na busca pelos sintomáticos respiratórios não foram determinadas pelo nível de conhecimento acerca da tuberculose.

## INTRODUÇÃO

Controlar a tuberculose (TB) no meio social vem se tornando cada dia mais desafiador, perpassando pela pouca investidura das equipes de saúde para identificar os sintomáticos respiratórios (SRs) de interesse para o diagnóstico oportuno^(1-3)^. Nesse sentido, os agentes comunitários de saúde (ACSs) são fundamentais, uma vez que desenvolvem suas atividades laborais no território, em contato direto com as pessoas^(4,5)^.

Causada pelo *Mycobacterium tuberculosis* ou bacilo de Koch, a TB acomete, preferencialmente, os pulmões, sendo denominada TB pulmonar, mas também pode comprometer outros órgãos, caracterizando a TB extrapulmonar^(1)^. Constitui relevante problema de saúde pública, pois figura, no âmbito global, como a principal causa de morte por um único agente infeccioso, muito embora, durante a emergência sanitária provocada pela pandemia de COVID-19, a TB tenha ocupado o *status* de segunda principal causa de morte, ficando atrás apenas da COVID-19. Em um contexto marcado por determinantes e vulnerabilidades em saúde, isso ressalta que os esforços para controlar a TB são prejudicados por fatores culturais, econômicos, políticos, sociais, clínicos e assistenciais^(6,7)^.

A adesão ao tratamento é vital para alcançar a cura e interromper a cadeia de transmissão^(6)^. No entanto, como afirmam dados oficiais, seu controle efetivo ainda se distancia da realidade, pois estima-se que, no mundo, em 2023, a doença acometeu 10,8 milhões de pessoas e gerou 1,25 milhão de óbitos^(7)^. Analisando o território nacional, verifica-se que, no mesmo ano, 80.012 pessoas adoeceram e, em 2022, 5.845 morreram por TB, resultando nos coeficientes de incidência e mortalidade iguais a 37,0 casos/100 mil habitantes e 2,72 óbitos/100 mil habitantes. No estado do Pará, esses indicadores se agravam, pois, em 2023, 4.293 pessoas adoeceram, e em 2022, 341 morreram, com os coeficientes de 48,8 casos/100 mil habitantes e 3,9 óbitos/100 mil habitantes^(8)^.

Em 2014, durante assembleia promovida pela Organização Mundial da Saúde (OMS), aprovou-se a Estratégia pelo Fim da TB (*End TB Strategy*), no intuito de enfrentar a doença e eliminá-la até 2035^(9)^. Devido à experiência do Brasil com a implantação e operacionalização do Sistema Único de Saúde (SUS), e com as contribuições da Rede Brasileira de Pesquisas em Tuberculose (Rede-TB), a participação desse país é fundamental para alcançar a meta global. Assim, baseado nessa estratégia, o Plano Nacional pelo Fim da TB como Problema de Saúde Pública tem como metas até 2035: reduzir o coeficiente de incidência para menos de dez casos/100 mil habitantes; e reduzir o coeficiente de mortalidade por TB para menos de um óbito/100 mil habitantes^(2)^.

A identificação de casos ocorre mediante a investigação de pessoas com suspeita da doença, configurando um rastreamento sistemático, para o qual devem ser utilizados testes ou outros procedimentos simples e rápidos^(3)^. Esse rastreamento ocorre por meio da busca ativa, processo de identificação de pessoas com tosse persistente que ainda não acessaram o serviço de saúde e que, de acordo com sintomas, possam estar com TB pulmonar ou laríngea. É necessário avaliar a duração da tosse e considerar o risco de adoecimento e o acesso do indivíduo aos cuidados formais, visando encaminhá-lo para realizar o exame diagnóstico^(10)^.

Os SRs são descritos como aqueles que apresentam tosse com duração igual ou superior a três semanas, ressaltando que, na busca ativa, o período de duração da tosse deve considerar a população investigada. É necessário identificar o SR para que seja diagnosticado e inicie o tratamento oportuno, interrompendo, assim, a cadeia de transmissão^(3,11)^. Nesse contexto, os ACSs são atores sociais que desempenham importante papel nas práticas de saúde relacionadas à busca dos SRs, visto que rotineiramente estão na comunidade para manter o relacionamento entre o usuário e o serviço de saúde^(4)^.

As estratégias para buscar os SRs devem ser bem planejadas, sobretudo no âmbito da Estratégia Saúde da Família (ESF), durante as visitas domiciliares, ocasiões nas quais os profissionais devem dialogar com usuários e compartilhar as orientações necessárias para examiná-los cuidadosamente^(12)^.

Considerando a potencial contribuição dos estudos sobre conhecimentos, atitudes e práticas (CAPs) para consolidar programas e ações voltados à prevenção e ao controle da doença, e tendo em vista a importância e multiplicidade de atribuições dos ACSs nesse contexto, entende-se que os saberes desses profissionais e a maneira como atuam são fatores que podem interferir na qualidade e efetividade de suas ações e, consequentemente, nos indicadores da doença no âmbito local^(4,5,13)^.

Os estudos sobre CAPs utilizam método específico e são delineados com o objetivo de mensurar o que as pessoas sabem, sentem, pensam e como se comportam em relação a determinado assunto, neste caso, a TB^(13,14)^. Faz-se tal ponderação considerando que elas apresentam conhecimentos variados, atitudes que se diferenciam e práticas adotadas ao longo do tempo^(15)^.

Assim, esses estudos ajudam a planejar, executar e avaliar as ações de saúde, possibilitando identificar problemas, necessidades, barreiras e soluções para melhorar a qualidade e acessibilidade dos serviços, contexto no qual os gestores definem metas e prioridades, estimam custos e recursos disponíveis, e promovem ações de capacitação profissional. Além disso, contribuem com a produção de dados sobre doenças e agravos para suprir os gestores e suas equipes com informações necessárias à formulação de novas estratégias, ajudando a identificar fatores que influenciam determinados comportamentos, os quais, muitas vezes, não são conscientes pela maioria das pessoas^(3,13,15)^.

Em termos conceituais, o conhecimento diz respeito à compreensão sobre qualquer assunto; a atitude está relacionada aos sentimentos e às ideias preconcebidas; e a prática representa as ações que advêm do modo como o conhecimento e a atitude se expressam. Entender os níveis de conhecimento, atitude e prática favorece o processo de criação dos saberes, incrementando possibilidades para que os serviços se adaptem e atendam às necessidades individuais e coletivas^(14)^.

Considerando a relevância científica e social do tema para o campo da saúde, especialmente a saúde coletiva, investimentos nele podem revelar o imaginário e os comportamentos dos ACSs em relação à TB e aos SRs, contribuindo para fortalecer estratégias de detecção dos sintomáticos e identificação dos casos novos, para os quais deve-se indicar o tratamento de forma oportuna.

Assim, definiu-se como objeto deste estudo: os CAPs dos ACSs sobre a detecção de SRs. Para investigá-lo, foram construídas as perguntas: quais os CAPs dos ACSs sobre a detecção de SRs? Esses elementos apresentam associação entre os ACSs na detecção de SRs na Atenção Primária à Saúde (APS)? Portanto, inicialmente, declarou-se a hipótese de que os CAPs interferiam na detecção de SRs.

## OBJETIVOS

Analisar a associação entre conhecimentos, atitudes e práticas de agentes comunitários de saúde sobre a detecção de sintomáticos respiratórios na Atenção Primária à Saúde.

## MÉTODOS

### Aspectos éticos

As orientações da Resolução nº 466/2012 do Conselho Nacional de Saúde/Ministério da Saúde foram seguidas, obtendo-se a anuência institucional da Secretaria Municipal de Saúde de Santa Izabel do Pará e a aprovação do Comitê de Ética em Pesquisa do curso de graduação em enfermagem da Universidade do Estado do Pará, emitidas em fevereiro de 2022. Os participantes leram e assinaram o Termo de Consentimento Livre e Esclarecido (TCLE), manifestando seu aceite voluntário. Para resguardar o sigilo das suas identidades, lhes foram atribuídos códigos alfanuméricos, formados pela sigla ACS, seguida por número arábico sequencial.

### Desenho e local do estudo

Estudo de corte transversal, norteado pelo instrumento *STrengthening the Reporting of OBservational studies in Epidemiology* (STROBE)^(16)^.

Foi realizado em 18 Unidades de Saúde da Família (USF) e em uma Unidade de Saúde da Família Ribeirinha (USFR) do município de Santa Izabel do Pará, estado do Pará, Brasil, um dos oito municípios que compõem a Região Metropolitana de Belém. Distancia-se cerca de 46,6 km em relação à Belém, capital do estado^(17)^, apresentando área territorial de 717,662 km^2^ e população estimada em 73.019 habitantes, de acordo com o Censo Demográfico vigente do Instituto Brasileiro de Geografia e Estatística^(18)^. Compõe-se por vários lugarejos e três distritos: Americano, Caraparu e Santa Izabel (sede municipal)^(19)^.

Essas unidades ofertam serviços da APS, por meio de equipes multiprofissionais constituídas por enfermeiros, médicos, odontólogos, técnicos de enfermagem, auxiliares de saúde bucal e número variável de ACSs (em torno de sete ou oito por unidade)^(20)^.

### População e critérios de seleção

Participaram 129 ACSs, representando 90,2% dos 143 que atuavam no município durante o período de coleta dos dados, configurando uma amostra censitária. Optou-se por incluir os que estavam no pleno exercício de suas funções e atuavam profissionalmente há, no mínimo, três meses. Foram excluídos dois (1,4%) por estarem de férias, licença ou afastados de suas funções, independentemente da causa, e 12 (8,4%) recusaram participar, totalizando 14 (9,8%) perdas entre os elegíveis. Além disso, não houve desistências.

### Protocolo do estudo

A primeira autora visitou as unidades para detalhar o projeto aos enfermeiros coordenadores das equipes de saúde da família, no intuito de solicitar seu apoio na operacionalização da coleta dos dados. Assim, em cada unidade, agendou-se uma reunião com os ACSs, que ocorreu em sala reservada, no intuito de apresentar-lhes a proposta, esclarecer dúvidas e convidá-los a participar. Os que aceitaram permaneceram em sala para agendar um encontro individual, que ocorreu reservadamente nas unidades em que atuavam, zelando por sua privacidade, sem interferir na rotina assistencial.

Para evitar possíveis vieses, os encontros foram conduzidos pela autora, mestranda em enfermagem, devidamente capacitada em reuniões programadas para atividades de orientação e nos encontros regulares de um grupo de pesquisa sediado na instituição dos autores. Iniciaram com a apresentação do TCLE para detalhar o objetivo, os procedimentos, riscos e benefícios da pesquisa, visando obter o aceite formal dos participantes. Em seguida, aplicou-se um instrumento formulado pelos autores, com base nas perguntas do guia *Advocacy, Communication and Social Mobilization for TB Control* para pesquisas de CAPs sobre TB, elaborado pela OMS^(13)^, e nas orientações da cartilha sobre TB para ACSs, publicada pelo Ministério da Saúde^(5)^, procedimento similarmente adotado em outro estudo^(21)^.

No formato de questionário autoaplicável, esse instrumento se constituiu por 37 perguntas, distribuídas em quatro seções, das quais a seção I apresentava dez perguntas sobre as características sociodemográficas e ocupacionais dos ACSs, cujas alternativas eram de múltipla escolha com resposta única. Essas perguntas foram: 1) “Quantos anos você tem?”; 2) “Qual é o seu sexo?”; 3) “Qual é o nível de formação mais alto que você concluiu?”; 4) “Qual a sua religião?”; 5) “Qual a sua cor/raça?”; 6) “Qual o seu local de trabalho/unidade de saúde?”; 7) “Qual o seu tempo de atuação como ACS?”; 8) “Qual o número total de usuários que você acompanha?”; 9) “Quantas pessoas com TB você acompanha?”; 10) “Quando foi a última vez que você recebeu treinamento sobre TB?”.

Sobre o conhecimento geral acerca da TB, a seção II se constituiu por 14 perguntas fechadas: 1) “A TB é causada pela bactéria *Mycobacterium tuberculosis*?”; 2) “A TB é uma doença grave?”; 3) “A TB é um problema em seu país/região?”; 4) “O aperto de mão e o compartilhamento de pratos, talheres e toalhas podem transmitir TB?”; 5) “A TB é transmitida pelo ar quando uma pessoa infectada tosse ou espirra?”; 6) “Entre os sinais e sintomas da TB, podemos citar tosse que dura mais de três semanas, tosse com sangue, perda de peso, fadiga, dor no peito e febre no final da tarde?”; 7) “O diagnóstico de TB pulmonar é realizado principalmente através da baciloscopia?”; 8) “A baciloscopia é realizada através da coleta de duas amostras de escarro, sendo uma no momento da identificação do SR e outra na manhã seguinte?”; 9) “Cobrir a boca e o nariz ao tossir ou espirrar é uma das orientações que devem ser dadas ao paciente com TB?”; 10) “A TB tem cura?”; 11) “O tratamento da TB está disponível no SUS?”; 12) “O tratamento da TB tem duração de seis meses, sendo dois meses correspondentes à fase de ataque e os outros quatro meses à fase de manutenção?”; 13) “Todas as pessoas com TB devem realizar teste rápido para HIV?”; 14) “O SR é aquele que apresenta tosse com duração de três semanas ou mais?”.

Apresentava as alternativas “concordo totalmente”, “concordo”, “indeciso”, “discordo” e “discordo totalmente”. Baseando-se em escala de Likert, tinha um escore final com três categorias de conhecimento, sendo elas “adequado/bom”, “regular” e “não adequado/insuficiente”. Atribuiu-se a categoria “adequado/bom” quando o ACS respondeu “concordo” ou “concordo totalmente” para ao menos dez perguntas; a categoria “regular”, quando respondeu “concordo” ou “concordo totalmente” para cinco a nove perguntas; e a categoria “não adequado/insuficiente”, quando respondeu “concordo” ou “concordo totalmente” para menos de cinco perguntas.

Sobre as atitudes na busca pelo SR, a seção III contemplou cinco perguntas fechadas: 1) “Quando identifico um paciente com tosse há três semanas ou mais, investigo a TB?”; 2) “Encaminho os SRs para consulta com enfermeiro e/ou médico?”; 3) “Encaminho os SRs para realizar baciloscopia de escarro?”; 4) “Registro a busca ativa de SRs na Ficha de Visita Domiciliar ou no aplicativo e-SUS AB Território (caso tenha *tablet*)?”; 5) “Relato para o enfermeiro os SRs para que ele os registre no Livro de Sintomáticos Respiratórios?”.

Apresentava as alternativas “concordo totalmente”, “concordo”, “indeciso”, “discordo” e “discordo totalmente”. Tinha um escore final com três categorias de atitude: “adequada/boa”; “regular”; e “não adequada/insuficiente”. Atribuiu-se a categoria “adequada/boa” quando o ACS respondeu “concordo” ou “concordo totalmente” para quatro ou cinco perguntas; a categoria “regular”, quando respondeu “concordo” ou “concordo totalmente” para duas ou três perguntas; e a categoria “não adequada/insuficiente”, quando respondeu “concordo” ou “concordo totalmente” para nenhuma ou somente uma pergunta.

Referente às práticas na busca pelo SR, a seção IV contemplou oito perguntas de múltipla escolha com resposta única: 1) “Você questiona presença e duração de tosse durante a visita domiciliar?”; 2) “Como você orienta a população quanto à transmissão aérea da TB?”; 3) “Como você orienta a população quanto aos sinais e sintomas da TB?”; 4) “Como você orienta a população quanto às medidas de prevenção que podem ser adotadas?”; 5) “Você prioriza a busca ativa de SRs em seu dia a dia?”; 6) “Você ensina como coletar material para a baciloscopia de escarro?”; 7) “Você comunica o enfermeiro quando encontra algum SR?”; 8) “Você questiona presença e duração de tosse nos contatos de pessoas com TB?”.

Apresentava um escore final com três categorias de prática: “adequada/boa”; “regular”; e “não adequada/insuficiente”. Atribuiu-se a categoria “adequada/boa” quando o ACS respondeu corretamente sete ou oito perguntas; a categoria “regular”, quando respondeu corretamente quatro, cinco ou seis perguntas; e a categoria “não adequada/insuficiente”, quando respondeu corretamente nenhuma, uma, duas ou três pergunta(s).

Os dados foram coletados entre os dias 06 de abril e 07 de outubro de 2022, período que foi precedido pelo pré-teste do instrumento, realizado na penúltima semana de março daquele ano, com dez ACS que atuavam em Benevides, município vizinho e que apresenta características econômicas e sociodemográficas similares às de Santa Izabel do Pará, não sendo incluídos na amostra deste estudo. O pré-teste contribuiu para adequar o questionário e estimar o tempo de sua aplicação. Ressalta-se que, embora não tenha sido validado com especialistas e/ou com o público-alvo, esse instrumento permitiu alcançar o objetivo do estudo.

### Análise dos resultados e estatística

Os dados foram organizados em planilhas do *software Microsoft Office Excel^®^* (versão 2016) para realizar análise estatística descritiva, identificando as frequências absolutas e relativas do perfil sociodemográfico e ocupacional e das características referentes aos CAPs dos ACSs. Para analisar a associação entre o nível de conhecimento e as atitudes e práticas, aplicou-se o teste qui-quadrado, utilizando o *software* BioEstat (versão 5.3). Consideraram-se o nível de significância de 95% e o valor de p≤0,05. A opção pelo uso de ambos os *softwares* se alinha à rotina e às práticas de trabalho no grupo de pesquisa.

## RESULTADOS

Entre os 129 participantes, predominaram o sexo feminino (n=96; 74,4%), a faixa etária de 40 a 49 anos (n=54; 41,9%), a escolaridade inerente ao ensino médio completo (n=86; 66,7%), a religião católica (n=65; 50,4%) e a cor/raça parda (n=109; 84,5%), como demonstra a Tabela 1.

**Tabela 1:** Características sociodemográficas dos participantes (N=129), Belém, Pará, Brasil, 2022

Características	n	%
Sexo		
Masculino	33	25,6
Feminino	96	74,4
Faixa etária		
Menos de 30	10	7,8
30 a 39	29	22,5
40 a 49	54	41,9
50 ou mais	36	27,9
Escolaridade		
Ensino fundamental completo	3	2,3
Ensino médio completo	86	66,7
Ensino superior completo	39	30,2
Não informada	1	0,8
Religião		
Católica	65	50,4
Evangélica	49	38,0
Sem religião	10	7,8
Outra	5	3,9
Cor/raça		
Branca	7	5,4
Parda	109	84,5
Negra	13	10,1

Quanto às características ocupacionais, verificou-se que 55 (42,6%) atuavam profissionalmente há mais de 20 anos e que o número de pessoas com TB acompanhadas pelos ACSs era variável, prevalecendo aqueles que, durante a coleta dos dados, referiram não estar acompanhando pessoas com essa doença (n=102; 79,1%). A maioria (n=78; 60,5%) recebeu treinamento sobre TB há mais de cinco anos, e apenas cinco (3,9%) receberam no último ano, sendo importante destacar que oito (6,2%) afirmaram nunca ter recebido (Tabela 2).

**Tabela 2:** Características ocupacionais dos participantes (N=129), Belém, Pará, Brasil, 2022

Características	n	%
Tempo de atuação profissional		
Menos de cinco anos	6	4,7
Entre cinco e nove anos	20	15,5
Entre dez e 14 anos	25	19,4
Entre 15 e 19 anos	23	17,8
20 anos ou mais	55	42,6
Número de pessoas com TB acompanhadas por ACS		
Nenhuma	102	79,1
Uma	21	16,3
Duas	3	2,3
Três	0	0,0
Quatro	1	0,8
Cinco	0	0,0
Mais de cinco	2	1,6
Último treinamento sobre TB		
Há um ano	5	3,9
Há dois anos	2	1,6
Há três anos	5	3,9
Há quatro anos	16	12,4
Há cinco anos	15	11,6
Há mais de cinco anos	78	60,5
Nunca recebeu treinamento	8	6,2

*ACS - agente comunitário de saúde; TB - tuberculose.*

Sobre o conhecimento geral acerca da TB, 121 (93,8%) apresentaram conhecimentos alocados na categoria “adequado/bom”, não sendo identificada a categoria “não adequado/insuficiente”. Da mesma forma, a maioria (n=113; 87,6%) apresentou atitudes alocadas na categoria “adequada/boa”, não sendo identificada a categoria “não adequada/insuficiente”. Além disso, 54 (41,9%) apresentaram práticas alocadas na categoria “adequada/boa”, e 17 (13,2%), na categoria “não adequada/insuficiente” (Tabela 3).

**Tabela 3:** Mensuração de conhecimentos, atitudes e práticas dos participantes a respeito da tuberculose e do sintomático respiratório (N=129), Belém, Pará, Brasil, 2022

Seção	n	%
Conhecimento		
Adequado/bom	121	93,8
Regular	8	6,2
Atitude		
Adequada/boa	113	87,6
Regular	16	12,4
Prática		
Adequada/boa	54	41,9
Regular	58	45,0
Não adequada/insuficiente	17	13,2

A associação estatística entre conhecimentos e atitudes não foi significativa (p=0,2582), assim como a associação entre conhecimentos e práticas (p=0,8973), permitindo afirmar que as atitudes e práticas dos ACSs na busca pelos SRs não foram determinadas pelo nível de conhecimento que tinham acerca da TB.

A proporção de ACSs com conhecimentos adequados/bons e que tiveram atitudes adequadas/boas (n=107; 88,4%) foi aproximada dos que tinham conhecimentos regulares e apresentaram atitudes adequadas/boas (n=6; 75,0%). O mesmo foi observado em relação às práticas, pois a proporção de ACSs com conhecimentos adequados/bons e que tinham práticas adequadas/boas (n=50; 41,3%), de igual modo, foi aproximada dos que tinham conhecimentos regulares e apresentaram práticas adequadas/boas (n=4; 50,0%), como demonstra a Tabela 4.

**Tabela 4:** Associação entre conhecimentos, atitudes e práticas dos participantes (N=129), Belém, Pará, Brasil, 2022

Atitude	Conhecimento				Valor de *p* ^*^
	Adequado/bom	%	Regular	%	
Adequada/boa	107	88,4	6	75,0	0,2582
Regular	14	11,6	2	25,0	
**Prática**					
Adequada/boa	50	41,3	4	50,0	0,8973
Regular	55	45,5	3	37,5	
Não adequada/insuficiente	16	13,2	1	12,5	

* *teste qui-quadrado.*

## DISCUSSÃO

Os resultados indicam, sobretudo, que os conhecimentos e as atitudes dos ACSs foram considerados adequados, contexto em que as práticas foram regulares em sua maioria, demonstrando que suas atitudes e práticas não estavam alinhadas ao que sabiam. Assim, refutou-se a hipótese do estudo, visto que os CAPs não interferiam na detecção de SRs.

Majoritariamente, expressaram conhecimentos adequados sobre diferentes aspectos da TB, como agente etiológico, gravidade, magnitude no país e na região, forma de transmissão, sinais e sintomas, diagnóstico, tratamento, e necessidade de realizar teste para diagnosticar possível infecção pelo Vírus da Imunodeficiência Humana (HIV), demonstrando conhecimento acerca do conceito de SR. Consideraram-se adequadas as atitudes relacionadas à identificação do SR de interesse para o diagnóstico da TB, à tosse há três semanas ou mais, ao encaminhamento do SR para consulta com enfermeiro e/ou médico, ao encaminhamento para realizar baciloscopia de escarro, ao registro da busca ativa e ao registro no Livro de Sintomáticos Respiratórios.

Por sua vez, as práticas foram consideradas regulares, salientando que o perfil adequado de conhecimentos e atitudes não refletiu nas ações dos ACSs junto à comunidade. É possível inferir que isso ocorreu devido aos ACSs conhecerem a doença e as atitudes necessárias para exercer a profissão, mas desconhecerem suas atribuições diante dela, fato possivelmente explicado por três aspectos: diferença de conhecimentos sobre saúde entre profissionais que atuam em determinado contexto; atitudes que se diferenciam entre eles; e práticas adquiridas ao longo do tempo^(22)^.

Nas características sociodemográficas e ocupacionais, destacaram-se o sexo feminino, a idade igual ou superior a 40 anos, a escolaridade até o ensino médio completo, a religião católica, os autodeclarados pardos, a atuação por 20 anos ou mais na função e os que declararam ter recebido treinamento específico para TB há mais de cinco anos. A predominância do sexo feminino nesse público também foi identificada em diferentes estudos, assim como a idade em torno dos 40 anos ou mais^(4,21,23,24)^. Todavia, em outra pesquisa, os ACSs receberam pelo menos um treinamento nos últimos dois anos, e buscavam informações sobre TB com colegas de trabalho e em panfletos, textos da internet, rádio ou televisão^(21)^.

Entende-se que a ausência ou pouca disponibilidade de ações de educação permanente sobre TB repercute no nível de conhecimento dos ACSs, visto que, no cotidiano do trabalho em saúde, lidam com muitos desafios para responder às demandas do território, exigindo informações contextualizadas para ofertar condutas qualificadas^(25)^.

Nesse contexto, figuram como atores essenciais para manter a vigilância epidemiológica, o que necessariamente inclui a busca ativa dos SRs. Por isso, as ações educativas, direcionadas aos ACSs, constituem medidas importantes para agregar conhecimentos e contribuir para a formação do pensamento crítico-reflexivo, visando à maior capacidade de articulação dos saberes técnicos com os saberes populares, os quais circulam fortemente entre os ACSs e na comunidade. Isso é relevante porque a capacidade de articular diferentes saberes, principalmente no contexto comunitário, é determinante para identificar os SRs^(4,26)^.

Para além das capacitações, há que se destacar como importante elemento o tempo de atuação como ACS, fundamental para estreitar os vínculos no íntimo contato com os grupos humanos, mas que também pode configurar oportunidade para agregar, solidificar ou ressignificar conhecimentos adquiridos ao longo do exercício profissional^(26)^.

Apesar de, neste estudo, as associações entre conhecimentos e atitudes e entre conhecimentos e práticas não terem obtido significância estatística, é preciso refletir que, em certa medida, conhecimentos podem influenciar atitudes e práticas sobre determinando tema, como aponta a literatura científica^(4,27)^. Assim, práticas equivocadas no contexto profissional, que resultam de lacunas do conhecimento, podem comprometer o alcance de objetivos e metas, sendo que, no caso da busca ativa pelo SR, podem interromper a detecção precoce de casos novos de TB e, assim, manter a cadeia de transmissão^(28)^.

Quanto ao tratamento, identificou-se que nem todos sabiam que a duração, na rotina das unidades da APS, é de seis meses, sendo que os dois primeiros correspondem à fase de ataque e os outros quatro, à fase de manutenção, como orientado pelo Ministério da Saúde^(3)^. Da mesma forma, alguns desconheciam a recomendação ministerial sobre a necessidade de realizar teste rápido para detectar ou descartar infecção por HIV em pessoas com TB^(3)^. Mesmo não sendo a maioria, entende-se que, como o trabalho dos ACSs ocorre de forma individualizada no território, ou seja, cada ACS responsabiliza-se por acompanhar uma área adstrita, é preciso uniformizar conhecimentos para alcançar resultados satisfatórios em todo o município^(4)^.

Estudo realizado com ACSs na Província do Cabo Oriental, África do Sul, identificou equívocos relacionados à causa da TB, associando-a ao tabagismo e ao frio, o que constitui cenário preocupante, visto o papel que esses profissionais desempenham na linha de frente do compartilhamento de informações sobre a doença em toda a comunidade, podendo resultar em atraso na identificação de casos^(23)^. Em outro estudo, realizado nos dez distritos de Lesoto, também localizado no continente africano, o conhecimento dos ACSs sobre TB foi classificado como inadequado, pois desconheciam informações básicas, como sinais e sintomas, e o que deve ser valorizado após a alta medicamentosa com o término do tratamento, cenário no qual os autores refletiram sobre a necessidade de ofertar ações de educação permanente, embora os participantes não tenham relacionado o conhecimento inadequado à ausência de um programa de capacitação sistemática^(24)^.

Admite-se que a longa atuação profissional, evidenciada na maioria dos participantes deste estudo, provavelmente contribui para aprimorar conhecimentos e estimular o compartilhamento de informações entre os ACSs^(14)^. Atribui-se a isso os conhecimentos satisfatórios acerca da TB, identificados inclusive entre os que relataram não ter recebido treinamento. Contudo, é necessário monitorar para que não haja reprodução de informações equivocadas sobre a transmissão da doença ou quaisquer outros aspectos, visto que o entendimento deturpado pode induzir práticas deletérias^(11,28)^.

Entre as cinco perguntas sobre as atitudes diante do SR, o maior número de acertos se relacionou ao encaminhamento para consulta com enfermeiro e/ou médico, sem considerar a necessidade de pronto encaminhamento para realizar baciloscopia de escarro, reforçando a visão de que apenas o enfermeiro e/ou médico são responsáveis por determinadas atitudes. Na cartilha sobre TB para ACSs, publicada pelo Ministério da Saúde^(5)^, consta que esses atores também podem encaminhar SRs para baciloscopia de escarro, desde que o município permita, por meio de protocolo.

A maioria concordou com a atitude de relatar para o enfermeiro a existência de SRs em sua área adstrita, entendendo-a como adequada, pois possibilita que o enfermeiro registre no Livro de Sintomáticos Respiratórios, muito embora, segundo os participantes, tal ação não se concretize no município em que este estudo foi realizado.

Na prática relacionada à orientação da população quanto à transmissão aérea da TB, aos sinais e sintomas, e às medidas de prevenção que podem ser adotadas, a maioria afirmou realizar, com frequência, ações de educação em saúde na comunidade. Ainda assim, uma parcela respondeu que apenas fornece orientações quando o usuário pergunta, e outros responderam que o enfermeiro é quem deve orientar durante as consultas. Vale ressaltar que, de acordo com o Ministério da Saúde, essas orientações fazem parte das atribuições dos ACSs^(5)^, assim como dos demais membros da equipe multiprofissional, como enfermeiros, médicos e técnicos de enfermagem^(2,3)^.

Todos concordaram com a prática relacionada à priorização da busca ativa de SRs, pois é importante que os casos de TB sejam identificados precocemente. No entanto, uma parcela afirmou ter outras demandas mais importantes para solucionar, e alguns afirmaram buscar SRs apenas quando dispõem de tempo ocioso, permitindo inferir que, talvez, entre os ACSs, havia aqueles que ainda não compreendiam a importância da busca ativa na comunidade.

Na prática de orientar visando à boa coleta de material para a baciloscopia de escarro, o entendimento dos ACSs é que se trata de uma atribuição do enfermeiro e/ou de profissionais do laboratório onde o exame será realizado, omitindo importante função, pois, em que pese o enfermeiro ter papel relevante nesse processo, o ACS também é responsável por tais orientações^(5)^.

Dialogando com esses resultados, outro estudo identificou que a maioria dos ACSs informou buscar ativamente os SRs durante visita domiciliar e encaminhar os casos suspeitos à ESF^(21)^, como normatizado pelo Ministério da Saúde^(5)^. Entretanto, somente uma pequena parcela informou solicitar baciloscopia e fornecer orientações sobre a coleta de escarro aos SRs, sugerindo lacunas de conhecimento entre esses profissionais e/ou problemas relacionados à estrutura ou organização das ações de controle da TB no âmbito local^(21)^.

A prática relacionada ao exame de contatos se mostrou negligenciada, pois os ACSs expressaram atitudes nesse contexto apenas quando os contatos referiam algo suspeito, considerando tal investigação, muitas vezes, desnecessária. Contudo, a literatura demonstra que a investigação dessas pessoas é prioritária, devido ao risco de adoecimento dos contatos infectados pelo *Mycobacterium tuberculosis*, que constituem fontes potenciais de infecção e manutenção da cadeia de transmissão na comunidade^(3,12)^. Assim, as ações de vigilância dos contatos necessitam de especial atenção por parte dos gestores municipais, no sentido de estimular as equipes de saúde e prover as condições necessárias para realizar sistematicamente essa atividade^(2)^.

Ressalta-se que as práticas dos ACSs devem ser monitoradas pelo enfermeiro, no intuito de avaliar seus conhecimentos e definir possível plano educacional direcionado e contextualizado, pois, com conhecimentos adequados, aumentam-se as possibilidades de implementar estratégias efetivas^(26)^. No trabalho em saúde, as atividades educacionais constituem uma propositura defendida no meio acadêmico-científico, visto que os profissionais devem ser atualizados regularmente para responder, de modo satisfatório, às necessidades de saúde dos grupos humanos^(22)^.

### Limitações do estudo

Como limitação, admite-se que o estudo foi realizado em apenas um município paraense, demonstrando uma realidade locorregional específica. Todavia, seus resultados podem subsidiar análises comparativas com outros cenários, investigando CAPs entre ACSs e outros atores.

### Contribuições para as áreas de enfermagem, saúde ou políticas públicas

Na medida em que evidencia as características sociodemográficas e ocupacionais e os dados inerentes aos CAPs dos ACSs, este estudo contribui para que atores da comunidade acadêmico-científica e da gestão pública, que atuam nos campos da saúde e da enfermagem, conheçam tal realidade e possam nela intervir, propondo e implementando novas estratégias que ajudem a transformá-la, contexto no qual se destacam as políticas públicas direcionadas aos profissionais.

## CONCLUSÕES

Verificou-se que os CAPs dos ACSs não apresentaram associação estatística significativa entre si, mas trouxeram importantes reflexões acerca da atuação desses profissionais e do funcionamento dos serviços da APS para controlar a TB no município investigado, visto que, apesar da predominância de conhecimentos e atitudes adequados, as práticas foram consideradas regulares, configurando importante divergência.

Salienta-se que, para controlar efetivamente a doença, é preciso ampliar ou fortalecer o nível de conhecimento dos profissionais para qualificar e reorientar suas práticas na rede assistencial, visando aperfeiçoar as ações nos serviços de saúde e na gestão clínica, considerando que seus CAPs podem aperfeiçoar ou comprometer a qualidade das estratégias de controle desenvolvidas na APS.

Em vista disso, recomenda-se que outros estudos sejam realizados acerca do tema, sobretudo com ACSs em diferentes cenários dos territórios regional e nacional, possibilitando, por um lado, melhor compreender os fatores que determinam seus CAPs e, por outro, analisar comparativamente os dados que resultarão desses estudos.

## Data Availability

Os dados da pesquisa estão disponíveis no corpo do artigo.
